# The use of self-assembling peptides (PuraStat) for hemostasis in cervical endocrine surgery. A real-life case series of 353 patients

**DOI:** 10.1016/j.ijscr.2022.107072

**Published:** 2022-04-12

**Authors:** Y. Gangner, M. Bagot d'Arc, C. Delin

**Affiliations:** a Department of Visceral Surgery, Niort Hospital, Niort, France; b BluePharm Consulting, 17 rue Davioud, 75016 Paris, France

**Keywords:** Hemostasis, Self-assembling peptides, RADA16, Endocrine cervical surgery, Thyroidectomy, Case series

## Abstract

**Introduction:**

Cervical endocrine surgery is frequent and carries the risk of rare but potentially life-threatening bleeding complications. Energy-based devices for stopping bleeding are not always usable in contact with nerves or parathyroid glands. Topical hemostatic agents may be an additional resource. PuraStat™, made of the self-assembling peptide RADA16, forms a new category of topical hemostatic agents.

**Objective:**

To assess the performance and safety of PuraStat to achieve hemostasis in cervical endocrine surgery.

**Methods:**

A retrospective chart review over four years was performed on 353 patients undergoing thyroidectomy and/or parathyroidectomy by a single senior surgeon, using PuraStat at the end of surgery in contact with recurrent nerves and parathyroid glands.

**Results:**

353 patients (79.06% female, mean age 54 years) underwent surgery with six weeks follow-up visit. Three patients had revision surgery for hematoma within the first 4 h (0.84%), which is within the low ranges reported in the literature. There was no delayed bleeding after 24 h, and dysphonia was observed in 15 patients, more severe for 2 patients (one unilateral and one bilateral palsy), and transient for the other 13 patients suggesting no product-related damage to the recurrent nerves. Hypocalcemia with clinical signs were reported in 8 cases. There were no unexpected adverse events.

**Conclusion:**

This is the first report of the use of PuraStat in patients undergoing cervical endocrine surgery, showing high performance and safety in achieving hemostasis and in preventing delayed bleeding without damage to the recurrent nerves. Further randomized controlled studies are needed to confirm the results.

## Highlights

• Cervical endocrine surgery carries the risk of rare but severe bleedings.
• Topical agents are additional resource to perform hemostasis in contact with nerves
• PuraStat forms a new category of topical hemostatic agents.
• First series of 353 patients with PuraStat in cervical endocrine surgery
• High performance in achieving hemostasis without damages to the recurrent nerves

## 1 Introduction

Cervical endocrine surgery carries the risk of rare but potentially life-threatening bleeding complications [1]. Prompt hemostasis is crucial to maintain good visualization of the surgical field and prevent damage to structures such as parathyroid glands or laryngeal nerves [2]. Energy-based devices have improved the safety of the procedures however, their use is limited or even harmful when in contact with fragile tissue [2], [3]. Topical hemostatic agents to which Purastat belongs are an alternative to conventional methods but are often of biological origin with potential risk of inflammation/infection, and their preparation can be time-consuming [4]. When facing oozing suffused bleeds of undetermined origin, surgeons are looking for a simple, effective, and tissue-safe solution to apply before closure. PuraStat is a specific class of synthetic and ready to use topical hemostatic agents that we have been used since 2016 in our daily practice. We report the results of 353 consecutive patients having received PuraStat during cervical endocrine surgery.

## 2 Materials and methods

As per the Declaration of Helsinki, this study is part of the Research Registry of the Health Data Hub in France with 221463v0 as registry number. The study has also been registered in the Research Registry and the UIN is 7789. Patients were informed of the study. Informed consent and ethical approval were not required as this case series involved the retrospective use of previously recorded data.

It is a real-life single center retrospective case series conducted from July 2016 to November 2020 in the department of visceral surgery at the public Hospital of Niort, France, by a senior endocrine surgeon.

Consecutive patients over 18 years of age with thyroid or parathyroid pathology, whether benign or malignant and operated on with the use of PuraStat as sole topical hemostatic agent were included. Anticoagulant therapies had to be bridged or stopped, except for aspirin. Patients were discharged the day after surgery. Patient characteristics, indication for surgery, types of procedures and complications were recorded. A de-identified database on Excel file was created based on patient's documentation.

**Fig. 1:**
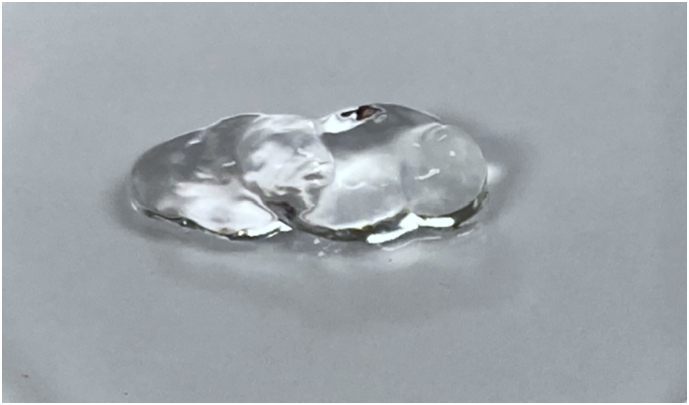
PuraStat™ RADA16 Self-Assembling Peptide Hydrogel © 3-D Matrix.

PuraStat (3-D Matrix Europe SAS, Caluire, France) is a colourless peptide hydrogel, ready to use, stored in the refrigerator. It is purely synthetic, made of 4 repeated amino acid sequences (aRginine - Alanine - aspartic aciD – Alanine) forming the RADA16 peptide capable of self-assembly at neutral pH (Fig. 1). PuraStat is a medical device, bioinert and without interaction with coagulation. It comes as a slightly viscous 2.5% aqueous solution with an acidic pH. In contact with blood or other physiological fluids, it rapidly neutralizes and forms a transparent hydrogel on the surface of bleeding tissue that mechanically blocks the blood flow, allowing hemostatic control [5]. PuraStat is indicated as a topical hemostatic agent during surgery, when hemostasis by ligation or conventional means is insufficient or impractical [6]. It is delivered in a 3 ml pre-filled syringe with a thin application nozzle.

The primary outcome was to evaluate the performance of PuraStat in controlling primary oozing bleeding in cervical endocrine surgery, by assessing the number of bleeding complications occurring within 24 h which required for revision surgery. Secondary outcomes assessed at the 6 weeks follow-up visit included incidence of delayed bleeding occurring ≥24 h postoperatively, tolerability of the product by the recurrent laryngeal nerves (RLN) assessed by the presence of dysphonia, and evaluation of other post-operative complications. Statistical analysis was performed using descriptive statistics for continuous variables, and categorical variables expressed as proportions. This case series has been reported in line with the PROCESS criteria [7].

## 3 Results

511 consecutive patients underwent open cervical endocrine surgery over the study period. 158 were not included as they did not meet the inclusion criteria. 353 patients undergoing 357 interventions were analyzed. There were 72 males and 281 females, with ages ranging from 17 to 86 years (median 54 years, mean ± SD 54 ± 14.09 years). Indications for surgery are illustrated in Table 1 and types of surgical procedures in Table 2. There were 336 thyroidectomies and 21 parathyroidectomies. Harmonic scalpel was used for dissection and Recurrent Laryngeal Nerves (RLN) were systematically monitored. Bipolar cautery allowed careful hemostasis. RLN were stimulated at the end and a small aspirating drain was placed in 67.51% of cases for 24 h. Just before closure, 3 ml of PuraStat was applied in a thin layer to the oozing tissue. PuraStat was used without drainage in 115 procedures (32.21%). The mean duration of surgery was 61.64 min (18–175, SD ± 19.87). A follow-up visit was performed on average 6.16 weeks after surgery for 352 cases. Five patients were lost to follow-up. Outcomes are presented in Table 3. The primary objective was to assess the performance of PuraStat to control oozing bleeding by measuring the number of bleeding complications that occurred within 24 h and required a revision procedure. Three patients (0.84%) had revision surgery for a hematoma in the first 4 postoperative hours. Another returned to theatre for neck swelling but no hematoma was found. No hemorrhagic event occurred more than 24 h after surgery. Therefore, the performance of PuraStat was 98.88% (353/357). No seroma nor surgical site infections were observed.

**Table 1:** Indications for surgery.

Number of operations	*N* = 357
Euthyroid multi-hetero nodular goiter	195 (54.62%)
Uninodal thyroids with compressive symptoms	93 (26.05%)
Uninodal or thyroid goiter in Hashimoto context	13 (3.64%)
Graves disease	23 (6.45%)
Papillary or epidermoid carcinoma	12 (3.36%)
Suspicion of Parathyroid adenoma	21 (5.88%)

**Table 2:** Types of surgical procedures.

Study cohort: number of procedures	N = 357
Primary surgery: 342	
- Lobo-isthmectomy	47 (13.17%)
- Total thyroidectomy	246 (68.91%)
- Total thyroidectomy + cervical node dissection	27 (7.56%)
- Thyroglossal cyst	1 (0.28%)
- Parathyroid surgery (2 with total thyroidectomy)	21 (5.88%)
Completion surgery: 15	
- Total thyroidectomy	10 (2.80%)
- Total thyroidectomy + cervical node dissection	4 (1.12%)
- Node dissection	1 (0.28%)

**Table 3:** Primary and secondary outcomes.

Primary performance outcome	
Hematoma within 24 h requiring revision surgery	3/357 (0.84%)


Secondary performance outcomes	
Hemorrhagic event >24 h postoperatively	0
Dysphonia at first follow-up visit	15 (4.20%)
Lack of response to electrical stimulation of laryngeal nerves stimulation at end of surgery	12 (3.36%)
Hypocalcemia within 24 h	4 (1.12%)
Tingling to fingers at first follow-up visit	8 (2.24%)


Safety outcomes	
Adverse events reported	0

At the follow-up visit, fifteen patients had mild or moderate dysphonia recovering for 13 of them, while one patient had bilateral palsy with respiratory discomfort that required a temporary tracheostomy, and another had an intra-tumoral nerve severed. Dysphonia was combined with a lack of response to electrical stimulation in 4 patients and with the presence of a significant malignant tumor in 11 patients. Twelve patients had no response to laryngeal nerve stimulation. Postoperative calcemia was measured routinely with hypocalcemia observed in 4 patients and tingling in the fingers in 8 (2.24%). Not all cases of hypocalcemia could be identified as some were performed after discharge. Histology was positive in 131/342 cases of primary surgery (38.30%). 96 showed low malignity grade or small size tumors (<10 mm) and 35 had a confirmed cancer with a total size ≥10 mm or with sign of severity. No other complications or unexpected adverse events were observed in the study.

## 4 Discussion

Thyroid surgery includes the risk of complications, of which bleeding is the most serious, ranging from the need for immediate reoperation to resulting in considerable damage to the laryngeal nerves and parathyroid glands [3]. In the literature incidence of hematoma varies from 0.3 to 2.1%, most occurring within 6 h and the re-intervention rate is 1.5%. Therefore, meticulous hemostasis is critical [8], [9], [10], [11], [12]. While energy devices are too dangerous for stopping small bleeding close to critical structures and clamp and tie are not possible, several studies have assessed the use of adjunctive hemostatic agents, PuraStat forming a new category [4], [7], [13], [14]. In our series, 3/357 hematomas (0.84%) required evacuation within 4 h of surgery, all after thyroidectomy with drainage. This rate of bleeding complications falls within the low ranges found in literature. There were no delayed bleedings, no seroma and no local surgical site infections, which are usually observed in 1 to 7% of cases [10], [13].

PuraStat has an acidic pH, rapidly neutralized and it was important to assess its safety in contact with RLN. According to the literature permanent dysphonia occurs in 0.5% to 5.0% of patients and transient injuries in 1% to 30% [15], [16]. In our study transient dysphonia was observed in 15 cases (4.20%). One nerve had to be severed, the others might have been stretched, devascularised or exposed to high temperatures making a neurotoxic effect of the product unlikely.

Immediate postoperative hypocalcemia ranges between 2.3 and 3.2% [10], [17]. In our series, 4 patients had hypocalcemia reported at the follow-up visit, one of them experienced a tetany attack and 8 reported tingling in fingers. This low rate (2.24%) is supportive of PuraStat being well tolerated when placed in contact to the glands parenchyma. In addition, 21 patients underwent an exploration of the parathyroid glands with 2 transient dysphonia as sole complications.

PuraStat has been evaluated for hemostasis efficacy and safety in many surgical specialties such as cardiovascular, gastrointestinal endoscopic and ENT surgery, with successful hemostasis rates ranging from 72.6 to 100%. No adverse events directly related to the device have been reported in these studies [7], [18], [19], [20], [21].

Results of our study demonstrate that PuraStat was easy to apply and effective in achieving hemostasis in absence of damage to the surrounding structures like laryngeal nerves and parathyroid glands. The additional cost of the device is comparable to that of similar products.

Our study has several limitations. Although all surgeries were performed by a single surgeon, it is retrospective and non-comparative. Certain variables that may influence outcome were not recorded, such as comorbidities, smoking status or concomitant treatment except anticoagulants. To our knowledge, this is the first series describing the use of PuraStat in open cervical endocrine surgery. Prospective randomized trials are warranted to confirm these findings and to assess the safety and effectiveness of PuraStat compared to the current standard care.

## 5 Conclusion

According to our experience PuraStat is easy to use and effective for hemostasis in open cervical endocrine surgery with less than 1% (0.84%) of postoperative bleeding, without damaging surrounding tissue. Further studies are needed to allow analyses with power.

## Provenance and peer review

Not commissioned, externally peer reviewed.

## Reporting checklist

The authors have completed the STROBE reporting checklist.

## Funding

This research did not receive any specific grant from any funding agency in the public, commercial or not-for-profit sectors.

## Ethical approval

This study is registered in the National French Registry of the Health Data Hub, was conducted in accordance with the current legislation on retrospective studies. Ethical approval was not required as this case series involved the retrospective use of already collected data.

## Consent

Informed consent was not required as per the applicable legislation on retrospective studies as this case series involved the retrospective use of already recorded data. Patients were informed about the study, and computerized treatment of their anonymized data.

## Author contribution

Conception and design: Y Gangner, M Bagot d'Arc, C Delin

Administrative support: All authors;

Provision of materials or patients: Y Gangner

Collection and assembly of data: M Bagot d'Arc, C Delin

Data analysis and interpretation: Y Gangner, M Bagot d'Arc, C Delin

Drafting the article: M Bagot d'Arc, C Delin

## Registration of research studies

Not applicable.

## Guarantor

Maurice Bagot d'Arc, MD.

## Declaration of competing interest

Yves Gangner has received consulting fees from 3-D Matrix Europe. Maurice Bagot d'Arc is a retired ENT surgeon working as a consultant for 3-D Matrix. Claudia Delin works as an independent consultant for 3-D Matrix.
